# Obesity prevalence, nutritional status, and physical activity levels in Turkish adults during the COVID-19 pandemic

**DOI:** 10.3389/fnut.2024.1438054

**Published:** 2024-08-06

**Authors:** Kezban Esen Karaca Çelik, María Morales-Suárez-Varela, Nazlı Uçar, Jose M. Soriano, Merve İnce Palamutoğlu, Murat Baş, Dilek Toprak, Ladan Hajhamidiasl, Özge Erol Doğan, Mert Doğan

**Affiliations:** ^1^Faculty of Health Sciences, Department of Nutrition and Dietetics, Acıbadem Mehmet Ali Aydınlar University, İstanbul, Türkiye; ^2^Department of Preventive Medicine and Public Health, Food Sciences, Toxicology, and Legal Medicine, School of Pharmacy, Area of Preventive Medicine and Public Health, University de Valencia, Valencia, Spain; ^3^Biomedical Research Center Network on Epidemiology and Public Health (CIBERESP), Institute of Health Carlos III, Madrid, Spain; ^4^Department of Medicine, Nutrition, and Diabetes, Boston University Medical Center, Boston, MA, United States; ^5^Food & Health Lab, Institute of Materials Science, University of Valencia, Paterna, Spain; ^6^Faculty of Health Sciences, Department of Nutrition and Dietetics, Afyonkarahisar Health Science University, Afyonkarahisar, Türkiye; ^7^Department of Family Medicine, Faculty of Medicine, İstanbul Atlas University, İstanbul, Türkiye; ^8^Department of Nutrition and Dietetics, Institute of Health Sciences, Acıbadem Mehmet Ali Aydınlar University, Istanbul, Türkiye; ^9^Department of Healthcare Services Ardahan, Vocation School of Healthcare Services, Ardahan University, Ardahan, Türkiye; ^10^Department of Physiotherapy and Rehabilitation, Faculty of Health Sciences, Akdeniz University, Antalya, Türkiye

**Keywords:** obesity, physical activity, international physical activity questionnaire, nutritional status, adults

## Abstract

**Background:**

Although nutritional status is influenced by multidimensional aspects encompassing physical factors, there is limited research on this complex relationship.

**Objectives:**

This study aimed to examine the interaction between physical activity level indicators and the nutritional status of adults in Türkiye.

**Methods:**

A total of 3,970 individuals aged 18 years or older, residing in Afyonkarahisar (Türkiye), participated in this study. Data were obtained through face-to-face interviews with a questionnaire, using simple random sampling among adults. One-day food consumption was recorded using a 24-h dietary recall (24HDR). Physical activity levels were assessed using the International Physical Activity Questionnaire (IPAQ).

**Results:**

This study comprised 3,970 participants: 2,087 (52.57%) men and 1,883 (47.42%) women. Among them, 32.64% smoked cigarettes, and 8.72% consumed alcohol. About 40% of adults were overweight, and 16.33% were obese. All participants had inadequate intake levels of potassium, calcium, and iron when compared to the recommended amounts. Physical activity levels showed a weak positive correlation with dietary energy, carbohydrates, protein, fat, and cholesterol, and a weak negative correlation with age, waist-to-hip ratio, and BMI of participants.

**Conclusion:**

This study provides insights into the importance of addressing both physical activity and nutritional status. It was found that the weekly duration of physical activity for women was lower than for men. As a result of the nutritional status evaluation, it was found that the daily energy intake of men was higher than that of women.

## 1 Introduction

Health-promoting behaviors, such as rational nutrition, physical activity (PA), a stable emotional state, and sufficient sleep, account for maintaining good physical health (1–3). Nutrition and PA are primary determinants of health and disease, associated with the risk of premature mortality, coronary heart disease, hypertension, colon cancer, type 2 diabetes, osteoporosis, and weight gain (4). Promoting PA and a healthy diet has the potential to substantially reduce the burden of disease and improve quality of life (5). Epidemiological studies have reported that decreased PA or increased sedentary activity is associated with higher health risks, such as low insulin sensitivity (6). Replacing 30 min of sedentary time with moderate to vigorous physical activity (MVPA) was associated with a 9.7% higher homeostasis model assessment of insulin sensitivity (7). Lack of MVPA is responsible for metabolic disorders such as metabolic syndrome. A reduction of 68–81% in the risks of lower abdominal obesity, hypertriglyceridemia, and low HDL cholesterol levels was observed in a study cohort using a SenseWear armband, spending more than 60 min per day in MVPA compared to those spending less than 30 min per day in MVPA (8).

Poor nutritional status among adults is associated with decreased immune function, higher healthcare costs, and longer hospital stays (9). Additionally, poor nutrition and low PA levels associated with low fruit and vegetable intake and excessive energy and fat consumption (10, 11). Brodney et al. (12) conducted a study with young women and men with different PA levels. Their results show that the diet of women and men with high PA was more balanced and characterized by higher vitamin intake compared to participants with low and moderate PA. Gacek et al. (13) found that the diet of young active women and men is often characterized by an insufficient number of meals during the day and too low intake of fish, vegetables, and fruit. Furthermore, the diet of physically active people includes excessive consumption of sweets, fast food, and sugary drinks, but insufficient water intake. For this reason, an unbalanced and low-variety diet in physically active people is related to an increased risk of inadequate vitamin and nutrient intake (14, 15).

The COVID-19 pandemic has profoundly impacted PA levels across diverse demographics, resulting in a widespread decline in PA. Specifically, older adults, particularly those residing in nursing homes, experience a negative impact on their PA levels (PALs), with community-dwelling residents demonstrating higher PALs than nursing home residents (16). Additionally, among students, the pandemic has led to a decrease in physical activity, with less than 70% meeting the World Health Organization’s (WHO) recommended daily physical activity levels by the WHO. This highlights the need to promote active lifestyles during pandemic-related restrictions as well as the importance of adhering to the WHO guidelines for physical activity (17). The COVID-19 pandemic has had a profound impact on the nutritional well-being of adults globally, resulting in alterations to dietary patterns, food security, and mental health. In the United Kingdom, the initial lockdown intensified food insecurity, particularly among lower-income groups who encountered difficulties in accessing food and exhibited reduced micronutrient intake, including riboflavin and vitamin B12, when compared to food-secure groups (18). In Thailand, lower-income older adults encounter significant nutritional challenges. During the pandemic, income inadequacy was found to have a positive correlation with both underweight and overweight status, illustrating the financial strain on nutritional well-being (19). To the best of our knowledge, there have been no studies with similar objectives conducted on the Turkish population.

Although nutritional status is influenced by multidimensional aspects encompassing PA and lifestyle, there is limited research on this complex relationship. Identifying the interaction of these factors in the nutritional status of Turkish adults can serve as a basis for developing nutritional education programs to improve nutritional status. The objective of this study was to determine the obesity prevelence, nutritional status, and PA levels of adult men and women in Türkiye. Accordingly, the research questions of this study are as follows:

- How common is obesity among adults in Türkiye when considering age groups and sex?
- What are the levels of physical activity among the adult population of Türkiye categorized by diverse age and gender groups?
- How does the nutritional status of Turkish adults differ by age and sex?
- Does a connection exist between physical activity, body mass index, and macronutrient consumption in the adult population of Türkiye?

## 2 Materials and methods

### 2.1 Study design

This study was conducted with participants aged 18–65 living in Afyonkarahisar, Türkiye, between January and April 2021. This study was a cross-sectional descriptive study. Ethical approval for the study was obtained from the Clinical Research Ethics Committee of Afyonkarahisar Health Sciences University (dated 05.05.2020 and No. 2020/5).

### 2.2 Participants, recruitment, and sample

A total of 3,970 individuals participated in this study. Data were obtained through face-to-face interviews with a questionnaire, selected by simple random sampling among adults. Data from individuals who voluntarily agreed to participate in the study were included in the analysis. Healthy individuals aged 18–65 were included in the study. Individuals who did not meet the inclusion criteria, had a mental disorder that could affect communication during the evaluation process, or had an active systematic disorder were excluded from the study. Additionally, participants’ personal information, names, and private information were not requested in the survey. A consent form prepared according to the Declaration of Helsinki was added to the questionnaire, and all participants signed this form.

GPower 3.9.1.4 software (G-Power, Universität Düsseldorf, Germany) was used for the *post-hoc* sample size analysis. The power of the study was 99.8% (decentralization parameter δ = 6.33/critical *t* = 1.96), assuming a Type I error probability (α) of 0.05 and the hypothesized association between physical activity level and food intake.

### 2.3 Data collection

No interventional procedure was performed on the subjects. To identify their anthropometric characteristics, the protocol required all questionnaires to be completed through interviews with subjects needing help with reading, writing, and understanding. General characteristics (age, sex, and education), some anthropometric measures [weight, height, Body Mass Index (BMI), waist circumference, hip circumference, and waist/hip ratio], food consumption, PA level, smoking, and alcohol consumption were questioned.

### 2.4 Assessments

#### 2.4.1 International physical activity questionnaire

The international physical activity questionnaire (IPAQ) was used to determine the physical activity level of the participants. The IPAQ provides researchers and professionals with an estimate of PA and sedentary behavior for adults aged 18–69 years across a variety of socioeconomic environments to determine PA levels (20). The validity and reliability of the scale were applied to adapt the Turkish version of the IPAQ in both the “long” and “short” forms by Sağlam et al. (21). With this questionnaire, individuals were asked about their PA over the past 7 days. They were questioned on the duration of vigorous PA (football, basketball, aerobics, fast cycling, weight-lifting, carrying loads, etc.), the duration of moderate PA (carrying light loads, moderate cycling, folk dancing, dancing, bowling, table tennis, etc.), daily duration of walking, and sitting time (in minutes). The criteria for physical activities were defined as in the IPAQ; they were performed for at least 10 min at a time. The scale consists of two types of scores: continuous and categorical. Continuous scoring entails calculating the met values for vigorous, moderate, and walking activities by multiplying these values by the duration and weekly frequency of each activity. The final score is obtained by summing the values obtained from each activity. Categorical scoring involves categorizing the physical activity level as low, moderate, or high (21).

#### 2.4.2 Antropometrics measurements

Participants’ body weight (in kilograms), height (in centimeters), waist circumference (in centimeters), and hip circumference (in centimeters) were evaluated and documented. The measurements were taken using standard equipment, including a SECA stadiometer, Tanita BC-480, and rigid measuring tape. All measurements were taken in accordance with the International Standards for Anthropometric Assessment (ISAK) guidelines, wearing light clothing and no shoes (22). Waist and hip circumferences were measured by a nutritionist using a rigid tape measure, and weight was measured with a scale (Tanita BC-480). Body Mass Index (BMI) was obtained by dividing the subject’s weight in kilograms (kg) by the square of their height in meters (m) [BMI = Weight (kg)/Height (m)^2^].

#### 2.4.3 24-h dietary recall

Food consumption records were collected by asking participants to recall their intake for the past 24 h, and were documented by a nutritionist (23). The energy intake, macro and micronutrient intake of each participant was subsequently analyzed using the BEBIS 9 (Nutrition Information System, İstanbul, Türkiye) program.

### 2.5 Statistical analysis

Descriptive statistical analysis was performed, and the response rate for each measure was evaluated. Data were analyzed using SPSS 26.0 (SPSS Inc., Chicago, IL, United States). Normality was checked using the Kolmogorov–Smirnov test. Chi square test was applied for nominal and ordinal data analyses. Mann Whitney U test and Kruskall Wallis test were used to analyze non-normally distributed data. Spearman test was used for correlation analysis. Results were presented as mean ± standard deviation or count and frequency, and *p* < 0.05 was considered significant. The correlation coefficient was accepted as 0.00–0.39 weak, 0.40–0.69 moderate, 0.70–0.89 strong, and 0.90–1.00 very strong (24).

## 3 Results

This study included 3,970 participants, with 2,087 (52.57%) men and 1,883 (47.42%) women. The general characteristics of the subjects are presented in Table 1. Age distribution was as follows: 18–29 years (43.8%), 30–39 years (23.45%), 40–49 years (16.30%), 50–59 years (11%), and 60–65 years (5.29%). The majority of participants were high school (33.88%) and university graduates (28.64%). Furthermore, 32.64% of the participants smoked cigarettes and 8.72% consumed alcohol. Statistically, all comparisons differed between sexes (*p* < 0.001).

**Table 1:** General characteristics of study subjects.

Parameters	Sex						*p*-value	*X* ^2^
	Men (*n* = 2087)		Women (*n* = 1,883)		Total			
	*n*	%	*n*	%	*n*	%		
**Age groups**								
18–29 years	970	24.43	772	19.45	1,742	43.88	<0.001^*^	31.32
30–39 years	445	11.21	486	12.24	931	23.45		
40–49 years	300	7.56	347	8.74	647	16.30		
50–59 years	248	6.25	192	4.84	440	11.08		
60–65 years	124	3.12	86	2.17	210	5.29		
**Education status**								
Not formal education	17	0.43	116	2.92	133	3.35	<0.001^*^	201.96
Literate	56	1.41	66	1.66	122	3.07		
Primary school	340	8.56	520	13.10	860	21.66		
Secondary school	192	4.84	181	4.56	373	9.40		
High School	833	20.98	512	12.90	1.345	33.88		
Higher Education	649	16.35	488	12.29	1.137	28.64		
**Smoking**								
Yes	1,021	25.72	275	6.93	1,296	32.64	<0.001^*^	530.18
No	1,066	26.85	1,608	40.50	2,674	67.36		
**Alcohol usage**								
Yes	284	7.15	62	1.56	346	8.72	<0.001^*^	132.39
No	1,803	45.42	1,821	45.87	3,624	91.28		

*n*, count; %, Frequency; ^*^p < 0.05.

The anthropometric measurements of the participants were organized according to age and sex in Table 2. The results indicated that men aged 39 years and younger had a lower mean age than women, whereas men aged 40–49 years had a higher mean age compared to women (*p* < 0.05). Furthermore, the study found that weight and height variables were different between the sexes in all age groups (*p* < 0.001). Specifically, men younger than 29 years of age had a higher BMI than women, and women aged 50 years and older had a higher BMI than men. Additionally, men in all age groups had a higher waist-to-hip ratio than women (*p* < 0.001).

**Table 2:** Age groups-based anthropometric measures of study subjects.

Parameters	Age groups	Sex				Difference between sex	
		Men (*n* = 2,087)		Women (*n* = 1,883)		*p*-value	*Z*
		X	SD	X	SD		
Age (years)	18–29	22.77	3.15	23.14	3.23	0.02^*^	−2.30
	30–39	34.13	2.79	34.58	2.81	0.03^*^	−2.14
	40–49	44.33	2.95	43.76	2.85	0.01^*^	−2.55
	50–59	54.05	2.8	54.03	2.73	0.97	−0.03
	60–65	62.18	1.85	62.25	1.94	0.78	−0.78
	Diff. between age groups	*p* = 0.001/H = 1693.6		*p* = 0.001/H = 1604.6			
Weight (kg)	18–29	79.11	12.93	61.01	9.77	<0.001^*^	−27.21
	30–39	79.75	11.32	69.29	10.47	<0.001^*^	−14.05
	40–49	82.15	10.8	73.27	10.68	<0.001^*^	−9.50
	50–59	80.67	11.5	76.38	12.68	<0.001^*^	−4.02
	60–65	81.63	12.72	78.13	13.01	0.02^*^	−2.24
	Diff. between age groups	*p* < 0.001/H = 26.53		*p* < 0.001/H = 501.71			
Height (cm)	18–29	177.55	7.71	162.95	7.23	<0.001^*^	−30.40
	30–39	174.31	6.97	163.62	6.11	<0.001^*^	−20.05
	40–49	173.85	6.22	163.61	7.7	<0.001^*^	−15.32
	50–59	170.45	6.76	159.44	6.34	<0.001^*^	−14.08
	60–65	170.13	6.25	159.16	6.74	<0.001^*^	−9.80
	Diff. between age groups	*p* < 0.001/H = 199.8		*p* < 0.001/H = 61.79			
BMI (kg/m^2^)	18–29	25.06	3.47	23.06	3.91	<0.001^*^	−12.31
	30–39	26.28	3.69	25.9	3.72	0.39	−0.86
	40–49	27.2	3.46	27.53	4.76	0.65	−0.44
	50–59	27.79	3.91	30.12	5.04	<0.001^*^	−5.60
	60–65	28.2	4.18	31.02	5.91	0.002^*^	−3.32
	Diff. between age groups	*p* < 0.001/H = 136.04		*p* < 0.001/H = 461.5			
WH ratio	18–29	0.89	0.07	0.79	0.1	<0.001^*^	−21.28
	30–39	0.89	0.07	0.84	0.1	<0.001^*^	−9.50
	40–49	0.9	0.09	0.85	0.09	<0.001^*^	−7.20
	50–59	0.92	0.09	0.86	0.09	<0.001^*^	−7.55
	60–65	0.94	0.1	0.86	0.09	<0.001^*^	−4.97
	Diff. between age groups	p < 0,001 / H = 33,41		p < 0,001 / H = 188,58			

BMI, Body mass index; WH Ratio, Waist/Hip Ratio; X, Mean; SD, Standard deviation; Z, Mann Whitney U Test; T, Independent samples T test; H, Kruskal-Wallis Test; ^*^p < 0.05.

The information contained in Table 3 represents the BMI classifications of participants based on their age and sex. As per the data, 7.18% of male adults and 9.12% of female adults were found to be obese. It was observed that approximately 40% of all adults were overweight and 16.33% of all adults were obese. Additionally, it was discovered that the age range with the highest obesity rate for male participants was between 40 and 49 years old, while for female participants it was between 60 and 65 years old. Notably, statistical differences were uncovered between the genders in adults aged 18–29, 50–59, and 60–65 years old (*p* < 0.05).

**Table 3:** Age groups-based BMI classification of the study subjects.

Age	BKI classification	Sex						*p*-value	*X* ^2^
		Men		Women		Total			
		*n*	%	*n*	%	*n*	%		
18–29	Underweight	18	0.45	64	1.61	82	2.07	<0.001^*^	102.38
	Normal	482	12.14	483	12.17	965	24.31		
	Overweight	439	11.06	191	4.81	630	15.87		
	Obesity	31	0.78	34	0.86	65	1.64		
30–39	Underweight	2	0.05	8	0.20	10	0.25	0.35	3.26
	Normal	164	4.13	177	4.46	341	8.59		
	Overweight	215	5.42	228	5.74	443	11.16		
	Obesity	64	1.61	73	1.84	137	3.45		
40–49	Underweight	0	0.00	4	0.10	4	0.10	0.12	5.85
	Normal	90	2.27	106	2.67	196	4.94		
	Overweight	137	3.45	138	3.48	275	6.93		
	Obesity	73	1.84	99	2.49	172	4.33		
50–59	Underweight	0	0.00	1	0.03	1	0.03	<0.001^*^	32.08
	Normal	60	1.51	27	0.68	87	2.19		
	Overweight	109	2.75	52	1.31	161	4.06		
	Obesity	70	1.76	112	2.82	182	4.58		
60–65	Underweight	0	0.00	0	0.00	0	0.00	0.009^*^	9.51
	Normal	26	0.65	10	0.25	36	0.91		
	Overweight	60	1.51	32	0.81	92	2.32		
	Obesity	38	0.96	44	1.11	82	2.07		
16–65	Underweight	20	0.50	77	1.94	97	2.44	<0.001^*^	96.21
	Normal	822	20.71	803	20.23	1,625	40.93		
	Overweight	960	24.18	641	16.15	1,601	40.33		
	Obesity	285	7.18	362	9.12	647	16.30		

BMI, Body mass index; BMI was classified according to WHO criteria, *X*^2^: Chi-Square Test; ^*^p < 0.05.

The data presented in Table 4 showed the weekly PA levels and amounts for participants grouped by age and sex. According to the findings, adult males aged 18–39 and 60–65 engage in greater amounts of physical activity than females (*p* < 0.001). Specifically, men aged 18–29 exhibited the highest level of vigorous PA (*p* < 0.001). Also, it was observed that 41.66% of the participants had moderate PA and 37% had low PA levels. The findings revealed that men had higher levels of PA women (*p* < 0.001).

**Table 4:** Age groups-based physical activity levels of the study subjects.

Physical activity	Age groups	Sex						Difference between sexes	
		Men		Women		Total		*p*-value	*Z*
		X	SD	X	SD	X	SD		
Vigorous (Min-MET per week)	18–29	1167.64	1909.48	515.75	1562.10	878.90	1793,07	< 0.001^*^	−11.11
	30–39	416.06	1546.09	630.22	1373.08	528.86	1460.61	< 0.001^*^	−4.09
	40–49	386.91	1453.11	962.06	2036.74	698.69	1814.59	< 0.001^*^	−4.66
	50–59	386.89	1576.04	299.09	1222.03	348.83	1432.36	0.59	−0.53
	60–65	561.34	2030.34	21.54	164.95	347.61	1600.79	0.003^*^	−3.00
	Diff. between age groups	*p* < 0.001/H = 226.93		*p* < 0.001/H = 30.53					
Moderate (Min-MET per week)	18–29	315.12	634.84	141.71	412.54	238.31	1688.63	< 0.001^*^	−8.38
	30–39	300.19	972.72	219.73	564.38	257.81	554.21	< 0.001^*^	−4.06
	40–49	304.63	861.54	256.83	632.62	278.72	785.15	0.21	−1.27
	50–59	203.30	762.65	167.61	622.58	187.83	745.93	0.33	−0.96
	60–65	75.63	191.69	134.36	384.91	98.88	704.75	0.69	−0.39
	Diff. between age groups	*p* < 0.001/H = 94.88		*p* < 0.001/H = 27.21					
Walk (Min-MET per week)	18–29	900.53	870.61	869.64	911.70	886.96	888.76	0.04^*^	−2.09
	30–39	946.65	1069.28	771.76	890.66	855.48	983.57	0.06	−1.88
	40–49	895.88	947.83	799.99	910.03	844.61	928.27	0.27	−1.10
	50–59	938.04	1008.61	836.41	1002.48	894.41	1006.03	0.32	−0.99
	60–65	740.08	827.65	581.83	687.89	678.36	778.19	0.02^*^	−2.32
	Diff. between age groups	*p* = 0.15/H = 5.26		*p* = 0.054/H = 7.65					
Total (Min-MET per week)	18–29	2278.19	2359.12	1333.70	1863.24	1870.76	2205.29	< 0.001^*^	−10.33
	30–39	1539.86	2378.29	1503.66	1520.99	1513.93	2023.21	< 0.001^*^	−1.91
	40–49	1485.34	2281.07	1869.57	1690.77	1643.00	2374.99	0.41	−0.82
	50–59	1309.89	2156.59	1057.27	1201.45	1320.20	2006.99	0.11	−1.58
	60–65	1302.44	2307.56	706.67	1070.09	897.46	1891.71	0.03^*^	−2.17
	16–65	1838.63	2362.02	1419.78	1641.78	1641.78	2169.65	<0.001^*^	−6.19
	Diff. between age groups	*p* < 0.001/H = 130.14		*p* < 0.001/H = 27.43					
Physical activity categories		*n*	%	*n*	%	*n*	%	*p*	*X* ^2^
Not reported		66.00	1.66	87.00	2.19	153.00	3.85	<0.001	37.89
Low		724.00	18.24	749.00	18.87	1473.00	37.10		
Moderate		868.00	21.86	786.00	19.80	1654.00	41.66		
High		429.00	10.81	261.00	6.57	690.00	17.38		
Total		2087.00	52.57	1883.00	47.43	3970.00	100.00		

X, Mean; SD, Standard Deviation; Z, Mann Whitney U Test; X^2^, Chi Square test; H, Kruskal-Wallis Test. ^*^p < 0.05.

The energy and macronutrient intakes of the participants are presented in Table 5. The approximately 1,975 ± 977, 1,787 ± 660, 1,653 ± 524, 1,651 ± 578, and 1,553 ± 530 kcal were the mean energy intakes of participants grouped by age. It was discovered that male adults aged 18–29, 40–49, and 50–59 years had higher energy intake compared to females (*p* < 0.05). In terms of carbohydrate intake, the participants’ approximate intake was 242 ± 123, 222 ± 92, 205 ± 78, 207 ± 83, and 196 ± 80 kcal, respectively. It was determined that men aged 18–59 years consumed more carbohydrates than women. Furthermore, it was found that men consumed more protein and fat than women, and the amount of protein and fat consumption decreased with age. Additionally, it was found that men aged 18–29 consumed more fat than women (*p* < 0.001).

**Table 5:** Self-reported energy and macro nutrient intakes of the study subjects.

Parameters	Age groups	Sex						Difference between sex	
		Men (*n* = 2,087)		Women (*n* = 1,883)		Total (*n* = 3,970)		*p*-value	*Z*
		X	SD	X	SD	X	SD		
Energy (kcal)	18–29	2104.54	980.83	1812.27	950.19	1975.02	977.94	0.001^*^	−8.01
	30–39	1793.12	563.34	1782.42	738.33	1787.53	660.17	0.11	−1.62
	40–49	1716.1	545.34	1598.45	500.56	1,653	524.69	0.002^*^	−3.07
	50–59	1702.92	592.9	1584.73	553.59	1651.35	578.41	0.03^*^	−2.15
	60–65	1552.71	544.1	1555.63	513.72	1553.91	530.62	0.8	−2.54
	Diff	*p* = 0.001/H = 46.61		*p* = 0.01/H = 11.03					
CHO (g)	18–29	256.26	123.16	224.65	121.78	242.25	123.52	0.001^*^	−6.39
	30–39	230.29	86.25	214.73	97.31	222.17	92.47	0.002^*^	−3.14
	40–49	212.68	73.85	200.17	81.68	205.97	78.33	0004^*^	−2.87
	50–59	214.99	87.27	196.7	77.13	207.01	83.4	0.03^*^	−2.14
	60–65	196.4	84.33	195.72	73.95	196.12	80.06	0.98	−0.02
	Diff	*p* = 0.001/H = 28.96		*p* = 0.09/H = 6.43					
CHO %	18–29	49	9.77	49.36	10.15	49.16	9.94	0.19	−1.31
	30–39	51.59	9.64	48.04	10.78	49.74	10.4	0.001^*^	−5.49
	40–49	50.53	8.44	50.43	10.56	50.48	9.63	0.45	−0.75
	50–59	50.73	9.41	50.73	10.1	5073	9.71	0.48	−0.69
	60–65	50.27	10.1	51.55	7.8	50.8	9.23	0.62	−0.49
	Diff	*p* = 0.001/H = 29.99		*p* = 0.001/H = 17.49					
Protein (g)	18–29	83.12	53.57	69.05	50.79	76.89	52.81	0.001^*^	−8.14
	30–39	64.44	25.77	68.14	37.16	66.37	32.26	0.53	−0.62
	40–49	63.63	24.29	56.99	22.02	60.07	23.32	0.001^*^	−3.3
	50–59	62.09	24.89	58.34	35.63	60.46	30.07	0.009^*^	−2.61
	60–65	55.78	23.5	53.49	18.45	54.84	21.56	0.62	−0.49
	Diff	*p* = 0.001/H = 34.62		*p* = 0.009/H = 11.53					
Protein %	18–29	15.17	4.08	14.65	4.45	14.94	4.26	0.001^*^	−3.60
	30–39	14.55	3.7	14.88	3.87	14.72	3.79	0.04^*^	−2.08
	40–49	15.21	3.83	14.53	3.71	14.85	3.78	0.01^*^	−2.43
	50–59	15.11	4.43	14.74	3.98	14.95	4.24	0.25	−1.15
	60–65	14.73	3.68	14.48	3.77	14.63	3.71	0.82	−0.22
	Diff	*p* = 0.002/H = 14.52		*p* = 0.20/H = 4.64					
Fat (g)	18–29	90.47	64.75	77.64	61.54	84.79	63.65	0.001^*^	−5.43
	30–39	66.37	25.36	76.1	46.4	71.45	38.12	0.22	−1.23
	40–49	64.75	26.13	61.22	25.85	62.85	26.02	0.08	−1.77
	50–59	63.11	25.55	60.52	29.09	61.98	27.15	0.11	−1.59
	60–65	58.56	21.31	57.35	23.11	58.06	22.02	0.59	−0.54
	Diff	*p* = 0.001/H = 38.96		*p* = 0.001/H = 17.09					
Fat %	18–29	35.83	8.99	35.99	9.16	35.9	9.06	0.89	−0.14
	30–39	33.86	8.95	37.08	9.83	35.54	9.55	0.001^*^	−5.80
	40–49	34.26	7.73	35.03	9.59	34.67	8.78	0.001^*^	−1.84
	50–59	34.16	9.01	34.53	9.16	34.32	9.07	0.70	−0.38
	60–65	34.99	8.71	33.98	7.17	34.58	8.11	0.64	−0.48
	Diff	*p* = 0.001/H = 24.24		*p* = 0.002/H = 15.14					
Cholesterol (mg)	18–29	347.31	332.89	269.27	279.45	312.72	312.67	0.001^*^	−6.31
	30–39	234.63	184.66	278.56	212.53	257.58	200.81	0.03^*^	−1.23
	40–49	221.83	154.96	216.94	138.28	219.21	146.16	0.97	−0.03
	50–59	216.61	153.27	216.12	184.53	216.4	167.43	0.41	−0.83
	60–65	224	156.53	202.63	136.21	215.25	148.58	0.44	−0.77
	Diff	*p* = 0.001/H = 50.2		*p* = 0.001/H = 17.25					

X, Mean; SD, Standard deviation; Z, Mann Whitney U Test; H, Kruskal-Wallis Test; ^*^p < 0.05.

The vitamin intakes of the participants are presented in Table 6. It was found that participants aged 40–65, men aged 30–65, and women aged 40–65 had insufficient levels of Vitamin E. Moreover, men aged 30–59 and women aged 40–49 were found to have inadequate levels of Vitamin C. Upon conducting sex-specific comparisons, it was determined that men aged 18–29 consumed higher amounts of Vitamins A, E, K, C, and B12 than women (*p* < 0.001).

**Table 6:** Vitamin intakes of the study subjects.

	Age groups	Sex									Difference between sex	
		Men (*n* = 2,087)			Women (*n* = 1,883)			Total (*n* = 3,970)			*p*-value	*Z*
		X	SD	RP %	X	SD	RP %	X	SD	RP %		
Vitamin A (μg)	18–29	969.9	523.1	107.77	942	550.1	134.57	957.5	535.2	119.64	0.04^*^	−2.085
	30–39	995.4	533.7	110.6	987.7	476.2	141.1	991.4	504.3	126.52	0.6	−0.53
	40–49	948.3	497.9	105.37	977.4	481.9	139.63	963.9	489.2	123.74	0.13	−1.5
	50–59	988.6	533.3	109.84	979.6	499.7	139.95	984.7	518.4	122.98	0.89	−0.135
	60–65	991.6	517	110.18	820.2	466.8	117.18	921.4	503	113.05	0.006^*^	−2.74
	Diff	*p* = 0.59/H = 1.91			*p* = 0.59/H = 11.95							
Vitamin E (mg)	18–29	29	35.7	193.55	21.9	32.9	145.85	25.9	34.7	172.41	0.001^*^	−6.69
	30–39	14.2	11	94.94	21.2	22.7	141.4	17.9	18.4	119.19	0.03^*^	−2.23
	40–49	12.7	7.7	84.38	14.6	9.6	97.45	13.7	8.8	91.39	0.03^*^	−2.18
	50–59	13.8	9.5	91.89	12.4	8.9	82.74	13.2	9.3	87.92	0.16	−1.4
	60–65	11.4	6.1	75.88	12.7	8.4	84.73	11.9	7.1	79.5	0.58	−0.55
	Diff	*p* = 0.001^*^/H = 55.17			*p* = 0.001^*^/H = 17.9							
Vitamin K (mg)	18–29	248.1	137.6	206.77	259.4	144.2	288.19	253.1	140.7	242.83	0.08	−1.73
	30–39	297	146.2	247.47	285.7	167.2	317.43	291.1	157.6	283.99	0.03^*^	−2.22
	40–49	303.9	145.6	253.21	276.4	185.1	307.12	289.1	168.4	282.12	0.001^*^	−3.67
	50–59	296.2	154.4	246.87	318.4	180.1	353.76	305.9	166.3	293.51	0.33	−0.97
	60–65	283.2	156.1	236.03	280.2	141.4	311.31	282	149.9	266.86	0.68	−0.41
	Diff	*p* = 0.001^*^/H = 71.56			*p* = 0.001^*^/H = 18.82							
Vitamin C (mg)	18–29	131.9	137.8	146.61	117.4	137.5	156.5	125.5	137.8	150.99	0.003^*^	−2.99
	30–39	78.1	47.9	86.79	127.3	118.3	169.68	103.8	94.9	130.11	0.001^*^	−5.43
	40–49	85.5	55.6	94.99	97.7	56.7	130.22	92	56.5	113.86	0.001^*^	−3.33
	50–59	87.1	72.8	96.75	94.9	56.8	126.47	90.5	66.3	109.72	0.01^*^	−2.49
	60–65	93.1	57.6	103.44	83.6	57.6	111.48	89.2	57.7	106.74	0.14	−1.46
	Diff	*p* = 0.001^*^/H = 24.28			*p* = 0.001^*^/H = 22.88							
Vitamin B12 (μg)	18–29	17.5	28	727.16	11.6	24.7	414.89	14.9	26.7	590.13	0.001^*^	−7.26
	30–39	4	5.8	165.04	11.4	19	407.9	7.9	14.8	292.38	0.001^*^	−3.3
	40–49	5	11.3	207.79	8.2	13	292.26	6.7	12.3	253.67	0.29	−1.06
	50–59	7.6	17	317.87	6	10.9	213.02	6.9	14.6	272.42	0.63	−0.48
	60–65	3.9	5	160.84	4.2	7.5	150.27	4	6.1	156.66	0.28	−1.07
	Diff	*p* = 0.001^*^/H = 82.65			*p* = 0.001^*^/H = 7.25							

X, Mean; SD, Standard deviation; Z, Mann Whitney U Test; H, Kruskal-Wallis Test; RP, Receiving percentage according to daily recommendations. ^*^p < 0.05.

The data on the intake amounts of potassium, calcium, and iron for the participants is presented in Table 7. According to the findings, all participants had inadequate intake levels of these nutrients when compared to the recommended amounts. In terms of sex-based comparisons, it was observed that men aged 18–29 and 40–49 consumed more potassium than their female peers. Furthermore, men aged 40–49 consumed more calcium, and men aged 18–29 consumed more iron than women (*p* < 0.001).

**Table 7:** Mineral intakes of the study participants.

	Age groups	Sex									Difference between sex	
		Men (*n* = 2,087)			Women (*n* = 1,883)			Total (*n* = 3,970)			*p*-value	*Z*
		X	SD	RP %	X	SD	RP %	X	SD	RP %		
Potassium	18–29	2228.3	1044.9	47.41	2084.4	1040.3	44.35	2164.5	1,045	46.05	0.002^*^	−3.153
	30–39	2148.5	842.8	45.71	2063.6	931.9	43.91	2104.2	891	44.77	0.16	−1.419
	40–49	2131.3	859.3	45.35	1930.4	858.7	41.07	2023.6	864.1	43.05	0.005^*^	−2.79
	50–59	2069.3	932.6	44.03	2144.7	965.7	45.63	2102.2	946.8	44.73	0.4	−0.84
	60–65	2005.3	884.1	42.67	2050.9	926	43.64	2,024	899.6	43.06	0.71	−0.375
	Diff	*p* = 0.5/H = 2.34			*p* = 0.5/H = 7.15							
Calcium	18–29	586.4	265.4	58.64	570.3	255.3	47.52	579.3	261	53.71	0.57	−0.56
	30–39	639.3	260.2	63.93	607	268.8	50.58	622.4	265.1	56.96	0.13	−2.48
	40–49	648.7	266.6	64.87	588.3	282	49.02	616.3	276.4	56.37	0.002^*^	−3.12
	50–59	625.7	284	53.05	657.7	293.2	54.81	639.7	288.2	53.82	0.15	−1.44
	60–65	619.8	280.8	51.65	532.4	261.5	44.37	584	275.8	48.67	0.02	−2.28
	Diff	*p* = 0.001^*^/H = 27.68			*p* = 0.001^*^/H = 15.9							
Iron	18–29	11.8	5.7	65.8	9.5	4	52.82	10.8	5.2	60.05	0.001^*^	−9.4
	30–39	11	4.6	61.05	10.8	4.7	59.73	10.9	4.7	60.36	0.64	−0.47
	40–49	10.4	4.2	57.78	11.7	5.3	65.18	11.1	4.9	61.75	0.01^*^	−2.5
	50–59	10.5	5.1	124.49	10.5	4.8	58.56	10.5	5	95.72	0.95	−0.62
	60–65	9.4	4.1	117.77	10.3	4.9	57.49	9.8	4.5	93.09	0.22	−1.24
	Diff	*p* = 0.001^*^/H = 27.26			*p* = 0001^*^/H = 42.53							

X, Mean, SD, Standard deviation; Z, Mann Whitney U Test; H, Kruskal-Wallis Test; RP, Receiving percentage according to daily recommendations. ^*^p < 0.05.

Relationship between physical activity, energy and macronutrients intake of the participants are presented in Table 8. It was found that participants’ physical activity levels had a statistically significant weak positive correlation with dietary energy, carbohydrates, protein, fat, and cholesterol, and a weak negative correlation with age, waist-to-hip ratio, and BMI. Additionally, it was found that individuals’ age had a weak positive correlation with waist-to-hip ratio (*p* < 0.001).

**Table 8:** Relationship between physical activity, energy, and macronutrients intake of the study subjects.

	PA level	BMI	Age	WH ratio	Energy	CHO	Protein	Fat	Cholesterol
PA level^r^	--								
BMI^r^	−0.06^**^	--							
Age^r^	−0.17^**^	0.40^**^	--						
WH ratio^r^	−0.13^**^	0.45^**^	0.18^**^	--					
Energy^r^	0.12^**^	0.11^**^	−0.12^**^	0.22^**^	--				
CHO^r^	0.11^**^	0.11^**^	−0.09^**^	0.20^**^	0.89^**^	--			
Protein^r^	0.14^**^	0.12^**^	−0.09^**^	0.25^**^	0.80^**^	0.66^**^	--		
Fat^r^	0.15^**^	0.10^**^	−0.11^**^	0.22^**^	0.79^**^	0.53^**^	0.65^**^	--	
Cholesterol^r^	0.16^**^	0.10^**^	−0.10^**^	0.24^**^	0.49^**^	0.34^**^	0.64^**^	0.56^**^	--

^r^Spearman’s Rho; CHO, Carbonhydrate; WH Ratio, Waist-Hip Ratio; ^**^p < 0.001.

## 4 Discussion

The specific aim of this study was to examine the interaction between physical activity indicators, lifestyle habits, and nutritional status of adults. To understand these relationships, this study identifies this situation. Nowadays, lower physical activity, socioeconomic and cultural problems, rapid urbanization, and changing dietary habits have altered the nature of people’s health issues. In this study, the physical activity levels and nutritional status of Turkish adults were assessed in a broad population.

According to the results of the 2018 Türkiye Demographic and Health Survey (TNSA), the majority of the population attended school, with 54% of men and 49% of women completing at least secondary education. The rate of people who completed at least high school was 29% for men and 21% for women (25). The results of this study indicated a negative association between physical activity and anthropometric measures. When the average BMI values were compared to education levels using data from the 2017 Türkiye Nutrition and Health Survey (TNHS), it was found that female BMI values decrease as education levels increase, while male BMI values do not significantly differ (26). In this study, it was found that 21.7% of the subjects had primary education, 9.7% had secondary education, 33.9% had high school education, and 28.6% had higher education. 3.4% of the subjects were not received formal education. Individuals with higher education levels had higher rates of physical activity. Therefore, it can be stated that education level may have a significant effect on physical activity, and obesity may increase with rising physical inactivity.

Global estimates of overweight and obesity levels (BMI ≥25 kg/m^2^), also called high BMI, according to data from the World Obesity Atlas 2023, indicate that more than 2.6 billion people could be affected in 2020 and more than 4 billion people by 2035. It is expected to increase from 38% of the global population in 2020 to more than 50% in 2035 (numbers do not include children under 5 years old). Only the prevalence of obesity (BMI ≥30 kg/m^2^) is expected to rise from 14 to 24% of the population during the same period, affecting approximately 2 billion adults, children, and adolescents by 2035 (27). Globally, in 2016, 23% of men and 32% of women over 18 were insufficiently physically active. In the last 15 years, levels of insufficient activity have not improved (28.5% in 2001, 27.5% in 2016). According to the 2022 WHO European Region Obesity Report, Türkiye is the country with the highest prevalence of obesity in the WHO European Region (28, 29). In Türkiye, 66.8% of the adult population is overweight, and 32.1% is obese. According to data published by the Turkish Statistical Institute (TUIK) in 2023, the obesity rate among obese individuals aged 15 and older in Türkiye was 20.2% in 2022. The results of this study showed that 7.18% of male adults, 9.12% of female adults, and 16.3% of all adults were found to be obese. Also, statistically significant negative weak relationship was determined between PA levels and BMI. When the relationship between age and physical activity level was examined, it was found that a negative weak correlation between PA level and age. These findings suggest that as individuals age, their physical activity levels tend to decrease, which may contribute to higher BMI and obesity rates.

Abu-Omar and Rütten (30) conducted a study on 29,193 people in 27 countries using the short IPAQ. They determined the subjects’ physical activity levels, indicating that leisure time physical activity can be an indicator of good health and supporting its use for monitoring purposes (30). Guthold et al. (31) compiled data from population surveys reporting the prevalence of insufficient physical activity, which included physical activity at work, at home, during transportation, and leisure time (i.e., not doing at least 150 min of moderate-intensity, or 75 min of vigorous-intensity physical activity per week, or any equivalent combination of the two). They included data from 358 surveys conducted in 168 countries, covering 1.9 million participants. The age-standardized global prevalence of insufficient physical activity was 27.5% (95% uncertainty interval 25.0–32.2) in 2016, with a sex difference of over eight percentage points (23.4%, 21.1–30.7, in men vs. 31.7%, 28.6–39.0, in women). Between 2001 and 2016, levels of insufficient activity remained stable (28.5%, 23.9–33.9, in 2001; non-significant change) (31). In this study, it was found that approximately 37% of all participants were low/inactive PA levels. The findings indicate many participants had low or inactive physical activity levels, posing a potential public health concern due to insufficient exercise. Additionally, women showed higher rates of inactivity, consistent with literature citing socio-cultural, behavioral, and physiological factors. Addressing these disparities is crucial for developing targeted interventions to promote physical activity and enhance health outcomes, especially in high-risk groups.

Burton and Turrell (32) reported that men have higher levels of physical activity than women. Despite the proven benefits of physical activity, 60% of adults in the United States do not engage in regular physical activities. Based on self-reported data from the Australian Bureau of Statistics National Health Survey, in 2014–2015, 56% of adults aged 18 and over were not sufficiently active (33). Similarly, another study conducted in England stated that men’s inactivity prevalence was lower than that of women (34). Strong evidence also demonstrates a dose–response relationship between sedentary behavior and all-cause mortality. Two meta-analyses were used to provide evidence of dose–response relationships between daily sitting or TV watching and all-cause mortality. Haase et al. (35) showed, in their study of university students from 23 different countries, that males were more physically active (36). However, Von Bothmer et al. (37), in their study of 479 university students in Sweden, evaluated physical activity levels, health habits, and motivation, finding no significant difference in physical activity habits between men and women. Nishida et al. (38) stated that women in Japan have recently become very interested in their body weight and physical activity, with the latter calculated as 73.6 and 56.3 min/day for men and women, respectively, showing that men engage in more physical activity than women. Furthermore, this relationship was statistically significant (*p* < 0.001) and was consistent with results from other similar studies. Findings of this research are consistent with prior studies. The elevated levels of physical activity (MET-Min) in males aged 18–29 as compared to older men and women of the same age can be attributed to several factors. Young men typically engage in active lifestyles, participating more in sports and exercise. Moreover, social interactions and cultural influences often encourage physical activity in this demographic. Work and school commitments, as well as leisure time management, also contribute to this trend. Further research should explore individuals’ health literacy and assess leisure activities and occupations to draw more accurate conclusions.

Baretta et al. (39) applied the short IPAQ to 597 adults in the Joacaba region, Santa Catarina, Brazil, finding low levels of physical inactivity and suggesting that this may help improve appropriate public health policies to increase regular physical activity. In current study, adults were evaluated. However, physical activity levels of different groups, such as children, elderly, workers from different companies, people living in rural areas or different geographical regions, housewives, etc., can be determined separately. The obtained data can be used to improve community health care programs and policies; especially, specific community health care policies can be developed to increase physical activity levels of groups with low physical activity levels.

A study from literature provides information on the importance of physical activity levels being affected by factors such as sex, age, socioeconomic status, education, biological, and psychological elements (40). For this reason, easy access to adequate facilities is important for physical activity. In our study, walking played a significant role in the total duration of physical activity, which may be because walking is the most convenient and economical physical activity. However, the reason vigorous physical activity duration is longer for men than for women may be because men engage in more vigorous physical activities, such as football and basketball, and have easier access to these activities. These results show that environmental factors affect physical activity levels. Therefore, efforts (education, facilities, financial support, etc.) to increase physical activity levels can be key guides for countries.

Daily energy intake and macro and micronutrient consumption are important for sufficient and balanced nutrition. Results from a study conducted at Türkiye, it was found that daily energy intake averages were 2,242 kcal for men and 1,649 kcal for women in the 19–30 age group; in the 31–50 age group, averages were 2,203 kcal for males and 1,638 kcal for females; in the 51–64 age group, they were 1,918 kcal for males and 1,533 kcal for females (25). In general, the results of current study showed that the average ages and energy intake are similar to the TNHS data. In addition, carbohydrate consumption is similar to the TNHS data for the 31–50 age group for both men and women. Protein consumption was found to be lower for women compared to men. However, when compared to TNHS data, protein consumption for males was lower. Fat consumption was observed to be lower for both males and females compared to TNHS data. It is thought that this may be due to different sociocultural characteristics and nutritional habits in the region.

According to the Recommended Dietary Allowance (RDA) values, the intake of vitamin A for adult males should be 900 μg/day, vitamin E intake should be 15 mg/day, vitamin K intake should be 120 μg/day, and vitamin C intake should be 90 mg/day. For adult females, vitamin A intake should be 700 μg/day, vitamin E intake should be 15 mg/day, and vitamin C intake should be 75 mg/day (41). In this study, it was observed that the daily intake of vitamin E, potassium, and calcium for both men and women were insufficient. For females, potassium and iron intake was considered very low. Low potassium intake may be due to low consumption of vegetables and fruits, and low calcium intake may be due to low consumption of milk and yogurt. Insufficient intake by women of childbearing age has been observed in some other studies conducted in Türkiye and constitutes an important public health problem. Therefore, the low intake of certain micronutrients may lead to new guidelines and care measures to improve quality of life. As indicated by Kalkan (42), the dietary habits of young adults in Türkiye were influenced by nutritional literacy, and therefore, much emphasis should be placed on increasing nutritional awareness among children, youth, and adults. In fact, dietary intake and biochemical parameters showed significant seasonal variations in Turkish elderly (43). Furthermore, a study conducted with Turkish university students demonstrated that there is more nutritional information about healthy dietary habits, adequate nutrient intake, and ideal body weight, reflecting the utility of food policy in Türkiye (44).

This research aimed to investigate the physical activity level and nutritional status of the Turkish population and to detect the relationship between them. The study’s strengths include a large sample size, which enhances the generalizability of the findings, and the examination of two fundamental factors that are critical to health, namely physical activity and nutritional status, and their relationship. However, the research has limitations. First, the 24-h food consumption record provides limited information about the nutritional status of individuals. Second, physical activity was assessed using a self-reported method, which may introduce partial bias depending on the participants’ tendency to provide accurate information. To overcome this limitation, it is recommended that technology-supported physical activity assessment tools, such as accelerometers and activity monitors, be used in future research. Finally, the study was conducted on a specific geographic region or demographic group, which may limit the generalizability of the results. Therefore, the authors suggest that future studies should examine the nutritional status and physical activity levels of children and elderly populations. In addition, continuing the research during the COVID pandemic reduces the generalizability of the data obtained.

## 5 Conclusion

With the rapid increase in the adult population, health promotion programs aimed at improving nutritional status can play an important role in health success. Identifying factors that influence nutritional status is essential when health professionals plan programs to help adults achieve and maintain optimal nutritional status. Based on the results of the research, the following conclusions were derived:

- The research shows that the prevalence of obesity is 16.33% for the Turkish population.
- Determining the elements that affect nutritional status is crucial for healthcare providers in devising initiatives aimed at enabling adults to attain and preserve optimal levels of nutrition.
- The results of this study indicate that physical activity and nutritional status ought to be addressed concurrently.
- The research demonstrated that females engage in physical activity for fewer weeks than males do.
- Consequently, the nutritional status evaluation revealed that males consumed more energy on a daily basis than females.
- The distribution of macronutrients was found to be disproportionate relative to daily energy consumption.
- Observations revealed that daily vitamin E, potassium, and calcium intake in both genders were inadequate, while the iron intake of the female group was lower than that of the male group.

The following recommendations were made based on the findings:

- Various forms of physical activity should be implemented and individuals should be encouraged to participate in a high level of physical activity to promote good health.
- Health promotion programs should be designed to address both physical activity and nutritional status simultaneously.
- Special attention should be given to ensuring balanced macronutrient distribution in daily diets.
- Efforts should be made to increase the intake of essential vitamins and minerals, particularly vitamin E, potassium, calcium, and iron.
- Programs should consider educational interventions to raise awareness about the importance of balanced nutrition and regular physical activity, especially targeting females and individuals with lower education levels.

## Data availability statement

The original contributions presented in the study are included in the article/supplementary material, further inquiries can be directed to the corresponding author.

## Ethics statement

The studies involving humans were approved by Afyonkarahisar Health Sciences University Non-Intervention Scientific Research Ethics Committee (dated 05.05.202 and No.2020/5). The studies were conducted in accordance with the local legislation and institutional requirements. The participants provided their written informed consent to participate in this study.

## Author contributions

KK: Conceptualization, Formal analysis, Investigation, Methodology, Visualization, Writing – original draft, Writing – review & editing. MM-S-V: Conceptualization, Formal analysis, Investigation, Methodology, Writing – original draft. NU: Conceptualization, Formal analysis, Investigation, Methodology, Writing – original draft. JS: Conceptualization, Formal analysis, Investigation, Methodology, Writing – original draft. Mİ: Conceptualization, Formal analysis, Investigation, Methodology, Visualization, Writing – original draft, Writing – review & editing. MB: Conceptualization, Formal analysis, Investigation, Methodology, Writing – review & editing. DT: Conceptualization, Formal analysis, Investigation, Methodology, Writing – original draft. LH: Conceptualization, Formal analysis, Investigation, Methodology, Writing – review & editing. ÖE: Conceptualization, Formal analysis, Investigation, Writing – review & editing. MD: Conceptualization, Formal analysis, Investigation, Writing – review & editing.
